# Imperial Caesar *Redivivus*

**Published:** 1909-11

**Authors:** 


### Imperial Caesar *Redivivus.*

The Autocrat of The Octopus is all-powerful. He and his cohorts got in their work on the great (?) Mississippi Valley Medical Association at St. Louis last month; whipped them into line; scared them to death. The meeting was almost a fizzle. Boasting that it is the next largest medical association in America—and *entirely independent*—there were less than two hundred in attendance. Why, the Texas State Medical gets together from five hundred to eight hundred almost any time. Dr. Lydston was gagged; not permitted to raise his voice in protest against the outrageous mismanagement of the affairs of the American Medical Association or plead for reforms in that great body in the interest of the rank and file. He was down on the program for a paper, "Despotism in Medical Organization," but he was not permitted to read it. We published this paper in October and are reproducing it in this issue to supply an unprecedented demand. Word was received at St. Louis from headquarters at Chicago that the paper must not be read, and the Executive Committee obeyed orders like a little man. But—a crowd of the appreciative and unterrified hired a hall and gave the valiant doctor an ovation. The daily press took it up, and the subject was better ventilated than if it had been read out loud in meetin'.

Toll! toll the bell, for Free Speech is dead! Ring out, ye iron bells. Chant, oh priests of The Oligarchy, a requiem for its soul! Dead! Dead! my Lords. No more shall the God-given right to be heard in protest against tyranny and oppression be exercised in medical societies, the privilege given to the humblest citizen by the Constitution of the United States. Cæsar sealed his ears against Casca's petition. But a Cassius and a Brutus arose and smote him! "We will meet again at Philippi"—in June.

(With apologies to Shakespeare.)

G. Frank Cassius (*loquitur*):

"Now in the name of all the gods at once,—upon what meat doth this our Cæsar feed, that he has grown so great? Age, thou art shamed!" I was born as free as Cæsar (but I was never a homeopath, nor conducted a Medical Institute with water cure and compound oxygen on the side). So were you (but you didn't advertise to cure every case, no matter of how long standing. * * * "And this man is now become a god, and Cassius is a wretched creature, and must bend his body if Cæsar but carelessly nod on him." * * * Ye gods! it doth amaze me, a man with such a record should so get the start of the medical world and bear the palm alone. Why, man, he doth bestride the medical world like a Colossus, and we petty doctors walk under his huge legs, and peep about to find ourselves dishonorable graves. * * * "It is not in our stars, but in ourselves, that we are underlings." * * *

"I speak this, perhaps, to *willing bondsmen,* * * * but every bondsman in his own hand *bears the power to cancel his captivity.*"

---

The Medical Association of the Southwest will meet in San Antonio November 9-11. It is expected that there will be a very large meeting and elaborate preparation to entertain the visitors has been made. San Antonio entertains delightfully and lavishly. Visitors are assured of a good time socially, and an intellectual feast at the sessions of the Association. The hotel accommodations are ample and first class; the railroad rates are low, and there is great interest attaching to everything in the historic old city. Let every doctor who reads this put down work —all work and no play, you know—and treat himself to the luxury of taking in this great event. It will be observed that the great Southwestern has no House of Delegates to lead the majority by the nose, blindly, but gives every member a voice, as they should do, and does the business through committees. We hope to see House of Delegates abolished in our Texas organization some day.

The following is a part of the preliminary program, received October 15. A complete program will be issued prior to the meeting, hence there is no necessity of publishing here and now (November 1) more than the schedule of the daily meetings, which follows. There will be an excursion to Mexico costing for railroad fare for round trip, good for twenty-five days, only $26.60, with stop-over privileges, and side trips can be made at special low rates. For further details address Dr. F. H. Clark, Secretary, El Reno, Okla. In the Section on Surgery there are thirty papers, Texas being represented by papers by many of our best known physicians.

PRELIMINARY PROGRAM.

Tuesday, November 9, International Club Rooms:

10:30 a. m.—General session and addresses of welcome and responses.

Meeting of Executive Committee.

2 to 6 p. m.—International Club rooms, scientific section work.

8 to 10 p. m.—International Club rooms, general session.

The President's annual address, Dr. Jabez N. Jackson, Kansas City, Mo.

Oration on Surgery, Dr. M. L. Harris, Chicago, Ill.

Oration on Medicine, Dr. Geo. Dock, New Orleans, La.

10 to 12 p. m.—Stag social and smoker.

Wednesday, November 10, International Club Rooms:

8:30 to 9:30 a. m.—General session.

9:30 to 12.—Scientific section work.

2 to 3 p. m.—The Medical Association of the Southwest meeting with the Fifth District Medical Association; an oration of general interest and the election of district officers.

3 to 6 p. m.—Scientific section work.

8:30.—Reception and banquet for the doctors and visiting ladies.

Thursday, November 11, International Club Rooms:

8:30 to 9:30 a. m.—General session, reports of officers and all committees.

9:30 to 11 a. m.—Scientific section work.

11 a. m. to 1 p. m.—Election of officers, selection of place for next meeting, etc., and adjournment.

3 to 5 p. m.—Automobile ride over city for the doctors and visiting ladies.

Committee meetings will be called as far as possible, so as not to conflict with the work of the scientific section.

Special excursion to "City of Mexico" will leave at 1 a. m., November 13. Sleepers will be ready for occupancy after 9:30 p. m., so that those desiring to do so may retire at the usual time.

---

THE APPEAL COURT has sustained the action of the Medical Examining Board in refusing to grant Morse a license because of unprofessional conduct. This settles the question, and the case of "Phenomenal Lafayette"—held up by the Bexar county court pending the decision of the Appeal Court in the Morse case—goes with it. Exit bloodless surgery and gallstones while you wait.

